# Dataset of spray deposit distribution in vine canopy for two contrasted performance sprayers during a vegetative cycle associated with crop indicators (LWA and TRV)

**DOI:** 10.1016/j.dib.2018.02.012

**Published:** 2018-02-13

**Authors:** Sébastien Codis, Mathilde Carra, Xavier Delpuech, Patrick Montegano, Henri Nicot, Bernadette Ruelle, Xavier Ribeyrolles, Blandine Savajols, Adrien Vergès, Olivier Naud

**Affiliations:** a IFV French Vine and Wine Institute, Montpellier, France; b Research Group ITaP, Irstea, Montpellier, France

## Abstract

In 2016, spray deposit measurements have been carried out according to ISO22522:2007 on a vine estate (Domaine Mas Piquet, 15 ha, Languedoc). On this estate, plots vigor ranges from low to medium compared to other vineyards. On 4 days (28th April, 25th May, 23rd June and 18th July 2016), spray deposition has been measured on 5 plots of different vine varieties chosen for their distinct vigor. Two different sprayers have been used: a low performance sprayer (pneumatic arch sprayer used every 4 rows) and a high performance sprayer (air assisted side by side sprayer). Spray deposition was measured using a tracer (Tartrazine E102) sprayed on sampling 40 cm² PVC collectors placed within the vegetation: on each plot, 4 trees have been sampled for each sprayer. On each tree, collectors were positioned on leaves within the canopy according to a profile perpendicular to the row, following a grid 20 cm high and 10 cm wide with one collector per pixel. A total amount of 3048 collectors have been analyzed individually. The dataset provide the normalized deposit expressed per unit of leaves area for one gram of tracer sprayed per hectare (unit: ng dm^−^^2^ for 1 g ha^−1^) on each collector. In addition, the dataset propose crop parameters measured manually on each sampled tree: inter-row distance, height, average of thickness in order to calculate two crop structure indicators: TRV (Tree Row volume) and LWA (Leaf Wall Area).

**Specifications Table**

**Table:** 

Subject area	*Agronomy*
More specific subject area	*Pesticide application, plant protection*
Type of data	*Table of spray deposits and crop parameters*
How data was acquired	Deposition: PVC collectors were positioned within the canopy. A Tartrazine (E102) water solution was sprayed on grapevines.
	After optical density measurement (*λ* = 427 nm) and considering the volume rate of mixture sprayed by hectare the normalized spray deposition was calculated (unit: ng dm^−2^ for 1 g ha^−1^) for each collector.
	Crop parameters: on each sampled grapevine for deposition measurement, crop parameters TRV and LWA have been measured.
Data format	*Spreadsheet (.csv format)*
Experimental factors	Data of deposition for 2 sprayers (High Perf and Low Perf) representing the range of spraying performance commonly observed in viticulture.
	Data for 5 plots within the same estate. Plots have been chosen for their distinct vigor.
Experimental features	*Data for 4 dates of treatment over the crop season: from F stage Baggiolini scale (4 to 5 leaves unfolded corresponding to the early first treatment) to L stage (berries beginning to touch, corresponding to the last treatment).*
Data source location	*Vineyard estate Mas Piquet (43°39'32.8"N 3°49'43.0"E) - Montpellier area, France*
Data accessibility	*Data are available with this article*.

**Value of the Data**

• These data are needed by epidemiologists to understand the links between plant characteristics, application technology and crop protection.
• These dataset is a necessary input for designing dose adjustment models based on indicators describing vegetation.
• A genuine database on distribution of pesticide deposition inside a wide range of vineyard canopies linked with plant characteristics.
• Data address two contrasted spraying technologies and five vine varieties during all vegetation cycle.
• This dataset describes for the first time the variability of plant protection product deposits within the canopy during the growing season on five plots of the same estate.

## 1 Data

Data provided in this article is a.csv file with 3048 lines which columns are:

A: Date of the trial.

B: Number of the treatment: from 1 (T1) to 4 (T4).

C: Plot name.

D: Vine variety on the plot.

E: Inter-row distance of the plot (in m).

F: Growth stage expressed in Baggiolini scale, from A to O.

G: Spraying modality:

– VC1/4hands: pneumatic arch sprayer used every 4 rows representing the most common practice in the French southern vineyard (Voûte Calvet^®^Ecoplus). The data is specific to the row sprayed by the hands.
– TPJ TXA: Air assisted side by side sprayer (Precijet, Tecnoma^®^) fitted with TXA800067VK hollow cone nozzles from Teejet® (angle 80°), Pressure 5 bars.

H: Tree number denomination.

I: Tree Row Volume (TRV) of the tree (expressed in m^3^/ha)

J: Leaf Wall Area (LWA) of the tree (expressed in m^2^/ha)

K: Height of the tree (in cm). This value is used to calculate the TRV and LWA

L: Tree average width (in cm).

M: Compartment number in thickness sense (from 1 to 6). N: Compartment denomination in the height sense (from A to J).

O: Width of the tree at the considered height (in cm).

P: Normalized deposit value on the collector (in ng dm^−^² for 1 g of tracer applied)

## 2 Experimental design, materials, and methods

### 2.1 Tree sampling

**Fig. 1:**
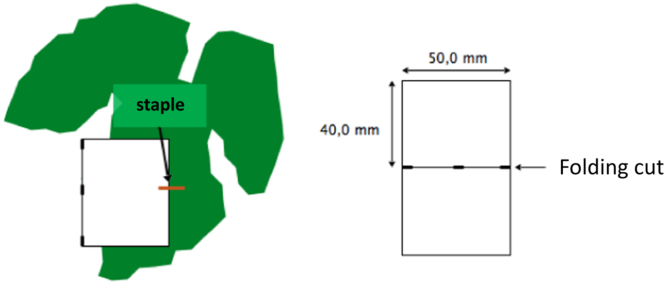
Collector shape used in the field to measure spray deposition.

For each 15 m row section, 4 trees were selected to be the most vigorous trees on this section. On each tree, collectors were positioned on leaves within the canopy. Dimension of each PVC collector was 8 × 5 cm (Fig. 1). Collectors had a section in their middle in order to fold them in two pieces of 4 × 5 cm and to staple on the leaves.

**Fig. 2:**
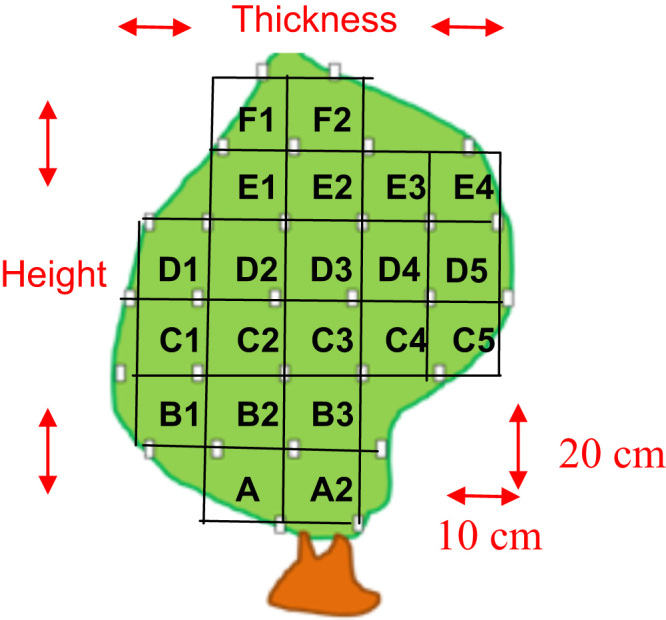
Grid used for deposit measurement on trees sampling.

Collectors were positioned according to a profile perpendicular to the row, following a grid 20 cm high and 10 cm wide with one collector per pixel (Fig. 2). The number of collectors depended on the height and the width of the tree (Table 1, Table 2).

**Table 1:** Number of heights considered to place the collectors within the canopy.

Height of foliage (cm)	Number of heights considered to place the collectors and heights denomination from the bottom up
[0; 20]	1 height: A
[20; 40]	2 heights: A and B
[40; 60]	3 heights: from A to C
[60; 80]	4 heights: from A to D
[80; 100]	5 heights: from A to E
[100; 120]	6 heights: from A to F
[120; 140]	7 heights from A to G

**Table 2:** Number of compartments in the depth depended on the width of vegetation measured at each height.

Width of the tree (cm)	Number of thickness considered to place the collectors and denomination
[0; 10]	1
[10; 20]	2: 1 and 2
[20; 30]	3: from 1 to 3
[30; 40]	4: from 1 to 4
[40; 50]	5: from 1 to 5
> 50	6: from 1 to 6

### 2.2 Sprayer settings on the field

**Fig. 3:**
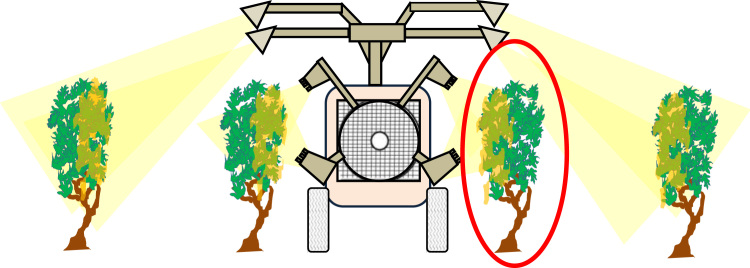
Pneumatic arch sprayer used every 4 rows: Voûte Calvet® Ecoplus. The data of deposition is relative to the row sprayed by the hands (in red circle).

**Fig. 4:**
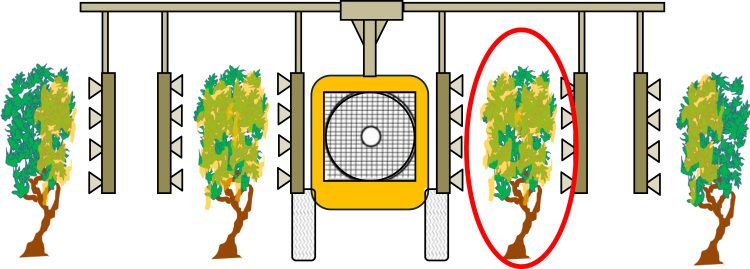
Air assisted side by side sprayer (Precijet, Tecnoma^®^) fitted with TXA hollow cone nozzles from Teejet®.

The two sprayers used are illustrated by Fig. 3, Fig. 4. A concentration of 10 g L^−1^ of tracer (Tartrazine, C_16_H_9_N_4_Na_3_O_9_S_2_) was used in the tank. For both sprayers, forward speed was 5 km h^−1^ and tractor's PTO on was set on 540 rpm. Calibration and nozzles caliber was chosen for both sprayers to spray around 150 L ha^−1^ of mixture for full growth stage i.e. when all outlets were opened. The numbers of active outlets was chosen in order to fit with the crop height and avoid losses above the canopy top. Before and after the spraying, the flowrate was measured by a flow measurement. Forward speed was measured during the spraying by measuring the time to cross the 15 m section where the 4 sampled trees were. The pressure of the liquid was 2.5 bars for the arch sprayer whereas the pressure was 5 bars for the air assisted sprayer. The reference of the hollow cone nozzles used during the trials was TXA800067VK, Teejet^®^.

### 2.3 Laboratory analysis

Each collector was rinsed with a controlled distilled water volume. Then, Tartrazine concentration was measured using a spectrophotometer (*λ* = 427 nm). Considering the volume rate of mixture sprayed by hectare, the concentration of the tracer in the mixture, the area of the collector and the forward speed, the normalized spray deposition (NorDeposit) was calculated (unit: ng dm^−2^ for 1 g ha^−1^) by the following formula:

$$\mathrm{NorDeposit}\;=\frac{Q}{\mathrm{VolRate}\times C}$$

With:

*C*: Concentration of dye in the tank (g l^−1^)

*Q*: Quantity of dye per unit area of collector (ng dm^−2^) expressed by:

$$Q=10^{9}\frac{c\times V_{d}}{S}$$

*S*: Area of the collector (in dm^2^)

*V*_d_: Dilution Volume (l)

*c*: Concentration of dye measured by spectrophotometry on a collector using the dilution volume (g l^−1^)

VolRate: Volume Rate of the sprayer (expressed in l ha^−1^) calculated by the following formula:

$$\mathrm{VolRate}=\frac{R\times 600}{F\times W}$$

With:

*F*: Forward speed of the sprayer (km h^−1^)

*W*: Width of plot sprayed in the field by the sprayer (m)

*R*: Flowrate of the sprayer (l min^−1^)

### 2.4 Measurement of crop parameters

Two crop indicators were calculated: LWA and TRV [1], [2], [3], [4], [5].

Leaf Wall Area (m² ha^−1^) was calculated with the following formula:

$$\mathrm{LWA}=\frac{2\times h\times 10000}{\mathrm{ir}}$$

With *h*: height of the canopy (m) and ir: inter-row distance (m). The *h* considered was the height that was taken into account for choosing the settings of the sprayer. On trimmed rows, *h* corresponded to the distance between the cord and the top of the vine (= the height of the topping).

Tree Row Volume (m^3^ ha^−1^) was calculated by the following formula:

$$\mathrm{TRV}=\frac{h\times t\times 10000}{\mathrm{ir}}$$

With *t*: average of thickness measures taken at each height (m). For each height, the thickness was measured without considering the branches that exceeded the plane of trellising.

The crop parameters *h* and *t* were measured on 4 trees of each plot that have been sampled for deposit measurement.

### 2.5 Analysis

**Fig. 5:**
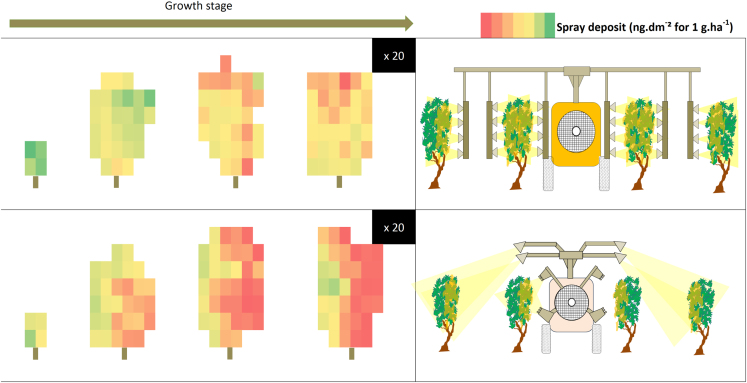
Deposit matrix of 1 tree for 2 sprayers at 4 growth stages.

Deposits matrix with a color scale were built to analyze variability of plant protection product deposits within the canopy during the growing season for two contrasted sprayers (Fig. 5).

## References

[1] del-Moral-Martinez I., Arno J., Escola A., Sanz R., Masip-Vilalta J., Company-Messa J., Rosell-Polo J.R. (2015). Georeferenced scanning system to estimate the leaf wall area in tree crops. Sensors.

[2] del-Moral-Martinez I., Rosell-Polo J.R., Company J., Sanz R., Escola A., Masip J., Martinez-Casasnovas J.A., Arno J. (2016). Mapping vineyard leaf area using mobile terrestrial laser scanners: should rows be scanned on-the-go or discontinuously sampled?. Sensors.

[3] Rosell J.R., Sanz R. (2012). A review of methods and applications of the geometric characterization of tree crops in agricultural activities. Comput. Electron. Agric..

[4] Walklate P.J., Cross J.V., Pergher G. (2011). Support system for efficient dosage of orchard and vineyard spraying products. Comput. Electron. Agric..

[5] Walklate P., Cross J.V. (2013). Regulated dose adjustment of commercial orchard spraying products. Crop Prot..

